# The impact on anatomical landmark identification after an ultrasound-guided palpation intervention: a pilot study

**DOI:** 10.1186/s12998-019-0269-4

**Published:** 2019-10-23

**Authors:** John Chinsuk Cho, Kenneth Reckelhoff

**Affiliations:** Department of Clinical Sciences, Parker University, 2540 Walnut Hill Lane, Dallas, TX 75229 USA

**Keywords:** Manual palpation, Chiropractic, Ultrasonography

## Abstract

**Background:**

To determine whether a discrepancy exists in identifying three musculoskeletal landmarks (medial meniscus, lateral malleolus and lateral epicondyle of the humerus) and whether ultrasound-guided (US-guided) palpation intervention can reduce that discrepancy and improve localization for chiropractic interns.

**Methods:**

Sixteen chiropractic interns were asked to identify three subcutaneous anatomical landmarks before/ after the intervention and at a 3-day follow-up. The intervention was a three-minute US-guided demonstration of the landmarks after the intern’s initial localization. The primary outcome measure was the change in distance between the intern’s landmark identification. Non-normal data were analyzed with the Friedman’s and Wilcoxon signed rank tests. Discrepancy between examiner-determined landmarks and intern-identified landmarks at the initial time point was assessed with a 1-sample Wilcoxon signed rank test.

**Results:**

All locations demonstrated an initial discrepancy between examiner-determined landmarks and intern-identified landmarks at the initial time point. Overall, a statistically significant difference was noted in the identification of the medial meniscus (*p* = 0.012) and lateral malleolus (*p* = 0.001), but not at the lateral epicondyle (*p* = 0.086). For the before and immediately after comparison, a significant improvement was found with the medial meniscus (*p* = 0.005) and lateral malleolus (*p* = 0.002). The 3-day post-intervention comparison found an improvement only for the lateral malleolus (*p* = 0.008).

**Conclusion:**

This pilot study demonstrated palpatory discrepancy at identifying all three landmarks. Our data suggests that US-guided palpation intervention seems to improve an intern’s ability to palpate two landmarks (medial meniscus and lateral malleolus) post-intervention.

## Background

Identification of topographical landmarks through manual palpation can be observed in different healthcare practices. Studies on reliability and validity of localizing various landmarks appear to have raised research interests over past decades [1–3]. Although manual palpation is an integral part of evaluating and delivering therapies to chiropractic patients, there are many studies demonstrating inaccuracy of landmark-driven palpation of various benchmark sites, such as using the posterior iliac crest to localize L4 spinous process [4–6] and the inferior angle of the scapula for identifying T7 spinous process [7]. In a review by Triano et al., several studies with high-quality evidence assessed validity and reliability of static palpation in the spine and pelvis. However, these studies conclude that the validity for localizing the site of care is in question. As for reliability, a favorable recommendation was confirmed, but with variable limitation depending on the target sites [8].

In interventional medicine, ultrasound-guided injection is widely done for several notable reasons. Most importantly, when ultrasound (US) guides the tip of the needle to the anatomic region of interest for administering medication, compared to the traditional ‘blinded’ injection method, improvement in prognosis is reported [9–12]. Understanding the nature of anatomical variations such as, but not limited to, the course of nerves, accessory nerves/muscles/ossicles and post-operative changes may be perceived as the reasons for improvement in diagnosis and therapeutic outcome for the patients receiving interventional therapy. Increased accuracy with manual contact points could result in more informed diagnostic decision-making and treatment success with manual therapies. It appears that US has not been used in the literature as a reference tool for assessing the identification of the landmarks in the extremities via palpation; likewise, US-guided intervention has not been examined for its potential impact on palpation. We feel that providing an educational intervention will strengthen the interns’ knowledge of neighboring anatomical landmarks through visual and tactile feedback.

The landmarks for the study were selected for two reasons. There are numerous palpatory studies done in the spine for accuracy of landmark-driven localization, but no studies of this nature have ever been reported for the extremities. Another reason for the selection is that each of the landmarks are commonly known sites of injury; meniscectomy is the most common orthopedic procedure performed in the United States [13], with the isolated medial meniscal tear more commonly seen than the lateral meniscal tear in patients older than 30 years old [14]. In the ankle, the ATFL is the weakest of the lateral ankle ligament complex and is most frequently injured [15]. Lastly, tennis elbow is a common injury of the proximal insertion of the extensor tendon at the latera epicondyle, and affects 1–3% of the population [16, 17].

The objective of the study was to compare the identification of three specific anatomical landmarks by manual palpation versus diagnostic US. Furthermore, through visualizing the landmark anatomy from a brief educational intervention under US (hence, we introduce the term US-guided palpation), we hypothesized that the intern’s ability to identify would improve. Our hypothesis is that an US-guided educational intervention will improve an intern’s ability to localize anatomical landmarks via palpation and provide further insight to palpatory training.

## Methods

We used a repeated-measures clinical trial study design with a convenience sample of 16 interns, who are interns treating patients at the university chiropractic clinics. Signed informed consents were obtained from all interns. Ethical approval was received by Parker University Institutional Review Board (IRB Protocol# A-00172).

Three anatomic landmarks were selected in the extremities where commonly encountered disorders occur: medial meniscus, lateral malleolus and lateral epicondyle of the humerus, which correspond to medial meniscal tear, anterior talofibular ligament sprain/tear, and tennis elbow.

### Study protocol

Each intern was asked to attend two sessions, distanced with 3 days, to measure for retention by the intern.

#### Initial session

On the initial session, the interns were requested to identify three landmarks on the subject model (male; BMI of 19.5) via manual palpation only. Verbal instructions for each of the landmarks were:

- Medial meniscus: Palpate and find the medial meniscus. You may move around the knee if you need to. Then place the Post-it® arrow in line with the long (circumferential) axis of the meniscus.
- Lateral malleolus: Palpate and find the lateral malleolus at the insertion of anterior talofibular ligament (ATFL), where you would find ATFL sprain/tear. You may move around the ankle if you need to. Then place the Post-it® arrow.
- Lateral epicondyle: Palpate and find the insertion of the extensor tendon at the lateral epicondyle, where you would find ‘Tennis elbow or lateral epicondylitis.’ You may move around the elbow if you need to. Then place the Post-it® arrow.

**Fig. 1:**
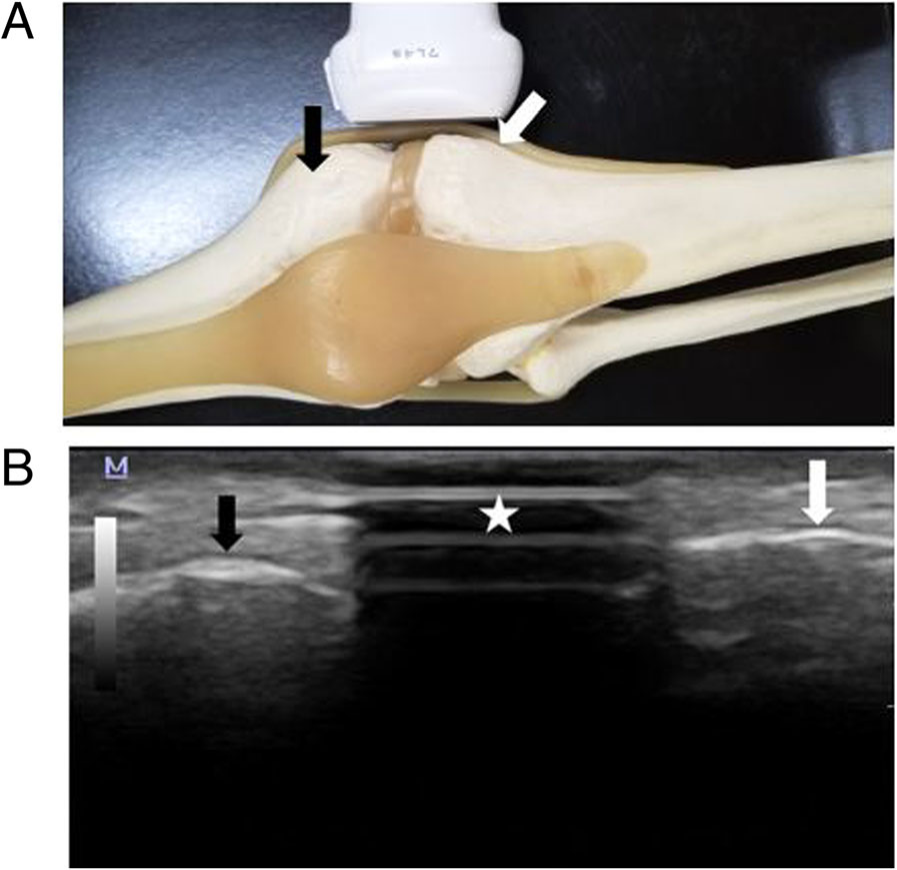
**a** Probe positioning for imaging the medial meniscus. The probe is placed over the medial meniscus with adjacent bony acoustic landmarks of the medial femoral condyle (black arrow) and tibia (white arrow). **b** Transverse (short axis) image of the medial meniscus demonstrating acoustic shadowing (star) from the Post-it® arrow placed over the medial meniscus, indicative of the intern’s localization being on the spot. Black and white arrows represent medial femoral condyle and tibia, respectively

**Fig. 2:**
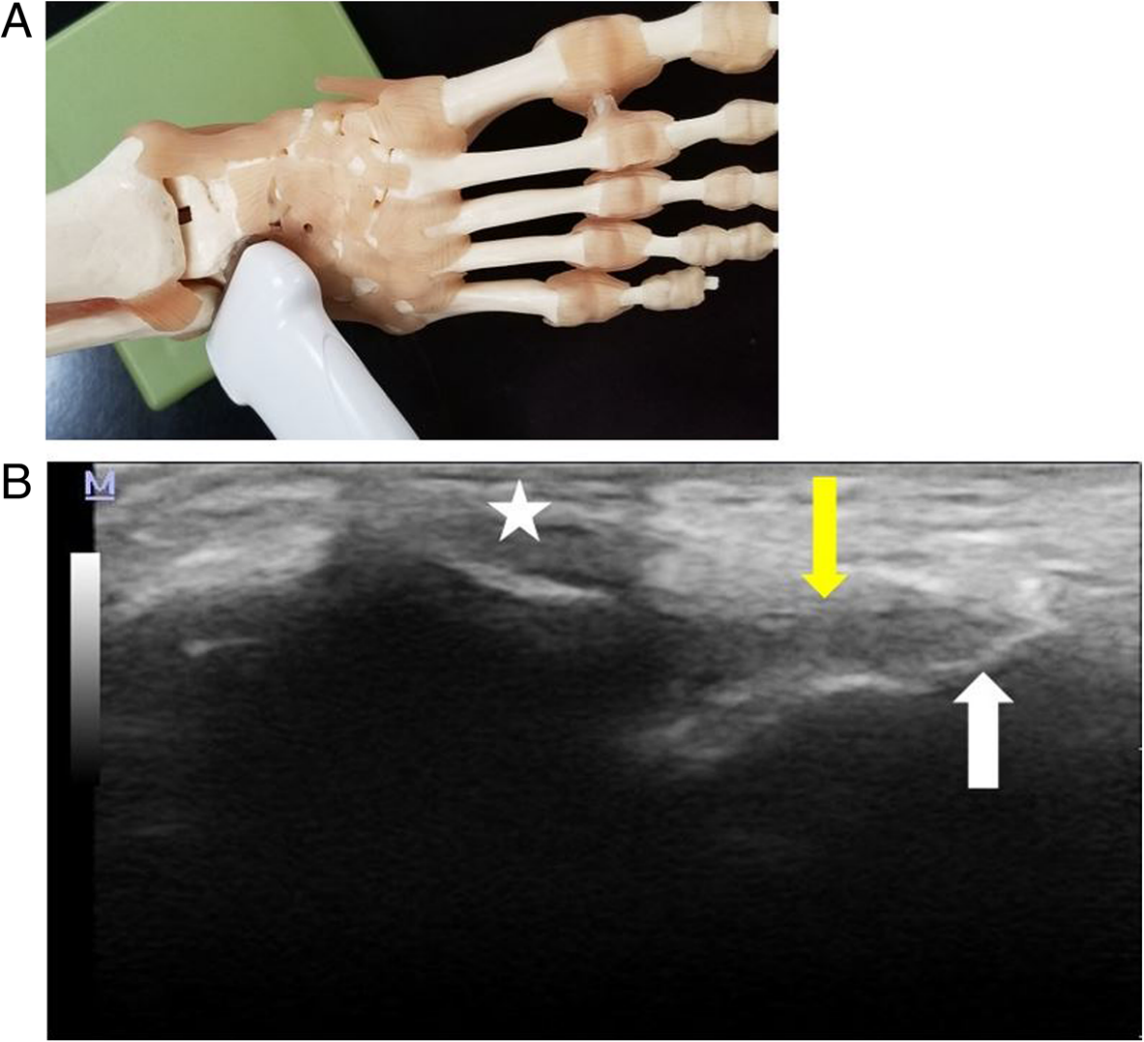
**a** Probe positioning for imaging the ATFL. The probe is aligned over the ATFL by visualizing the acoustic bony landmarks of the lateral malleolus and the talus. **b** Longitudinal image of the ATFL (yellow arrow) depicting acoustic shadowing (star) from the Post-it® arrow through the lateral malleolus. The entirety of the ATFL is visualized with the ligament inserting to talus (white arrow) distally

**Fig. 3:**
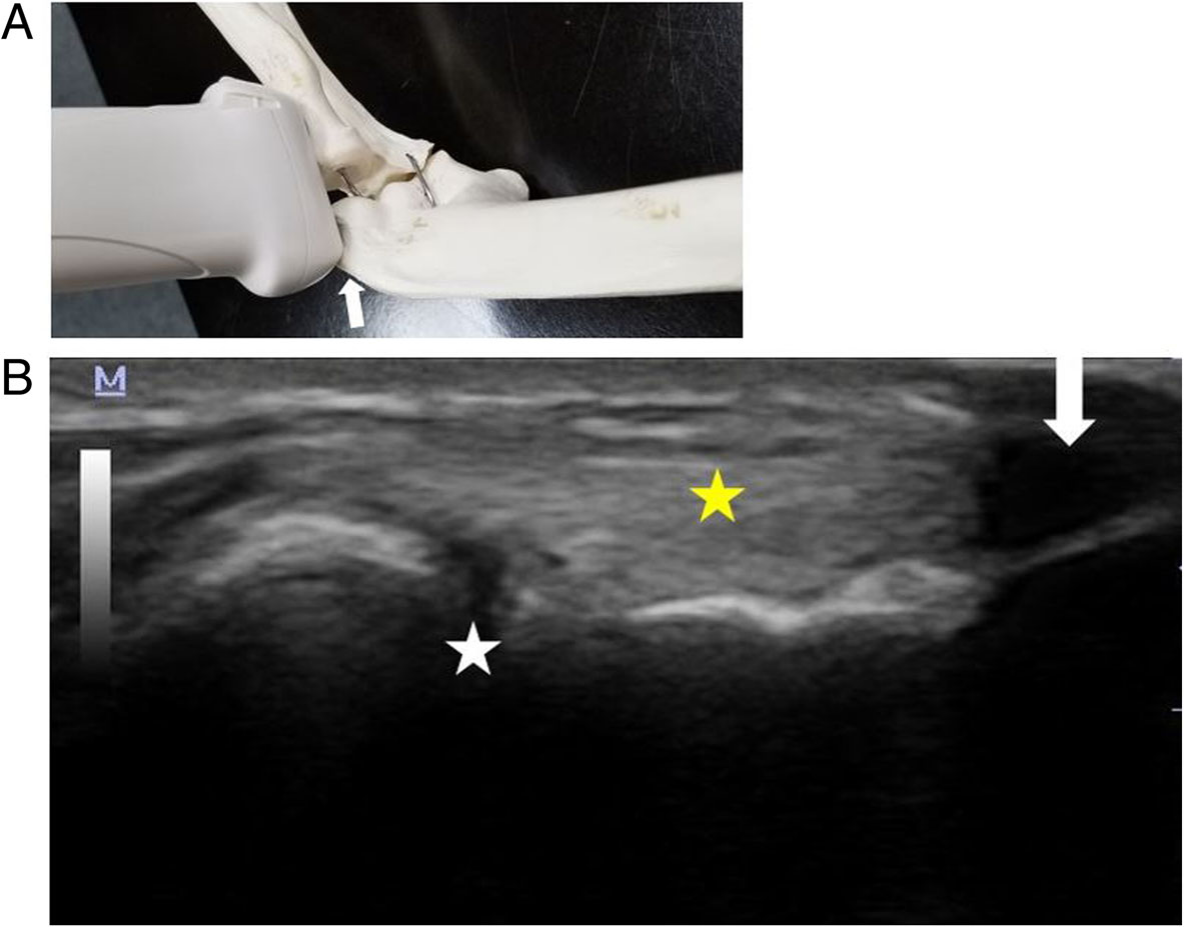
**a** Probe positioning for imaging the lateral epicondyle of the humerus (white arrow). While the insertion of the common extensor tendon is imaged, both the lateral epicondyle and the radial head is captured for consistency. **b** Longitudinal image of the lateral epicondyle demonstrating acoustic shadowing (white arrow) from the Post-it® arrow. For imaging reproducibility, the landmark for scanning lateral epicondyle includes radiocapitellar joint (white star) deep to the extensor tendon (yellow star)

After the manual palpation was complete, the examiner scanned the target site with US, then placed a different colored Post-it® arrow only if the landmark was not correctly identified by the intern. US was performed with a single unit, Mindray M5 ultrasound scanner (Mindray, Shenzhen, China), using a 7–12 MHz linear array transducer. All ultrasound scans and image recording were done by a single examiner (JCC) and verified by the co-investigator for agreement on the image. The examiner measured the distance between the 2 points of the Post-it® arrow with the subject model verifying the measurement (recorded in millimeters). Both of the investigators have an average of 7.5 years of experience in musculoskeletal (MSK) ultrasound and both are registered as MSK sonographers (RMSK®) by the Alliance for Physician Certification and Advancement. US procedures for each landmark were:

- Medial meniscus: The subject model was seated with the knee extended and the foot resting on a stool. The examiner first placed the transducer in short axis over the Post-it® arrow already placed by the intern. If the medial meniscus was correctly identified by the intern, which would have been demonstrated with acoustic shadowing (the Post-it® arrow prevents transmission of ultrasound waves through it) through the meniscus (Fig. 1), the distance measurement will be marked as ‘0 mm’ for being on the landmark. If any part of the Post-it® arrow (width = 12 mm) was placed over the meniscus, it was still considered as being on the landmark. If the intern’s Post-it® arrow was not on the medial meniscus, the examiner scanned away from the intern’s Post-it® arrow towards the hypoechoic medial meniscus, staying in the same plane. Once the medial meniscus was located, a different colored Post-it® arrow was placed by the examiner. The medial meniscus location is defined as the hypoechoic space that bisects the free edges of the femur and the tibia.
- Lateral malleolus: The subject model was seated with the knee extended and the foot resting on a stool. The ankle was in slight medial oblique position in order to expose the ATFL. If the intern’s placement of the Post-it® arrow revealed acoustic shadowing was visualized over any part of the lateral malleolus and the ATFL (Fig. 2), it was qualified as being on the landmark with a measurement recorded as ‘0 mm.’ If not, then under US, the examiner located the lateral malleolus where the ATFL inserts and placed the Post-it® arrow. The lateral malleolus location is defined as the most distal end of the fibula with attachment of the hyperechoic fibrillar ATFL between the fibula and the talus in view.
- Lateral epicondyle: For scanning the lateral epicondyle, the subject model was seated with the hands pronated and resting on the lap. If the intern’s identification of the landmark was not correct, then the examiner would locate the apex of the lateral epicondyle and place the Post-it® arrow over the apex of the lateral epicondyle. In order to qualify as being on the target site (i.e. measurement of ‘0 mm’), the two examiners (JCC and KR) looked for the acoustic shadowing of the Post-it® arrow overlapping both the lateral epicondyle and the extensor tendon or the acoustic shadowing on the extensor tendon that was agreed between the investigators to be abutting the lateral epicondyle (Fig. 3). The lateral epicondyle location was defined as the apex of the bony lateral epicondyle with the hyperechoic common extensor tendon and the radio-capitellar joint in view.

The interns were given 3 min of brief US-guided palpation intervention which entailed US-guided feedback to accurately understand the exact location of the landmarks. The primary investigator displayed subcutaneous landmarks visible under US that were adjacent to and inclusive of the landmark of interest. Furthermore, tactile characteristics and a systematic approach for identifying the landmarks were discussed during the brief educational intervention. The intern was then instructed to identify the same anatomical landmarks on the contralateral side of the body. The principal investigator then scanned the target site with the US, placed another Post-it® arrow (only if the intern’s identification of the landmark was not correct) and obtained a follow-up distance measurement.

#### Follow-up session (after 3 days)

The interns were provided with the same verbal instructions they received during the initial assessment to localize the three landmarks. Measurements were obtained for retention of the interns from the US-guided palpation training 3 days prior.

### Data

Statistical analyses were conducted using the IBM SPSS Statistics 19 software (Chicago, IL). Histograms were constructed to analyze for the assumption of normality, and the Shapiro-Wilk test was used, which revealed non-normal distributions and *p*-values less than 0.05. A 1-sample Wilcoxon signed rank test was used to analyze whether landmarks were correctly identified at the initial time point. Friedman’s test was used to analyze for differences within each landmark group. Post-hoc testing was carried out to identify for individual differences within each landmark using the Wilcoxon signed rank test. Six separate comparisons were made; therefore, the Bonferroni adjusted alpha level for post-hoc testing was *p* ≤ 0.0083 (0.05/6). Effect sizes were calculated for each instance and averaged to achieve a single estimate (*r* = 0.31) to be used for *post-hoc* power analysis. Achieved power was calculated using *r* = 0.31, Bonferroni adjusted α level = 0.0083 and *n* = 16 and was low at 6%.

## Results

A total of nine males and seven females in their final clinical year were recruited as interns. Descriptive statistics and 95% confidence intervals of the difference in distance (in millimeters) of each landmark by session/time are reported in Table 1 and reveal a general trend for improvement in distance over time. There was discrepancy between the examiner and interns at all 3 locations (adjusted alpha level = 0.17; medial meniscus, *p* = 0.005; lateral malleolus, *p* = 0.001; lateral epicondyle, *p* = 0.001) at the initial time point.

**Table 1:** Median (IQR) and mean (SD) difference in millimeters of each landmark by session/time

Location	Time*	Median (IQR) distance between	95% CI of median distance between	Mean (SD) distance between	95% CI of mean distance between
Medial meniscus	1	7 (13)	0–13	7.75 (7.46)	3.77–11.72
	2	0 (6)	0–7	2.56 (4.30)	0.27–4.86
	3	0 (17)	0–17	9.50 (13.02)	2.56–16.44
Lateral malleolus	1	18 (11.75)	8–21	15.75 (10.23)	10.30–21.20
	2	1 (7.5)	0–8	3.69 (4.90)	1.08–6.30
	3	2 (9.75)	0–10	4.81 (5.97)	1.63–7.99
Lateral epicondyle	1	12 (10.5)	5–16	12.63 (9.91)	7.34–17.91
	2	6 (11.5)	0–12	6.50 (6.63)	2.97–10.03
	3	0 (13.25)	0–14	6.25 (8.61)	1.66–10.84

*Time 1 = First session, before educational intervention

*Time 2 = First session, immediate post-intervention

*Time 3 = Second session, 3 days post-intervention

As shown in Table 2, there were statistically significant differences across the three time points (before educational intervention, immediately post-intervention and 3 days post-intervention) in the medial meniscus (χ^2^ (2, *n* = 16) = 8.773, *p* = 0.012) and lateral malleolus (χ^2^ (2, *n* = 16) = 14.25, *p* = 0.001), but not at the lateral epicondyle (χ^2^ (2, *n* = 16) = 4.9, *p* = 0.086) (Table 2).

**Table 2:** Results demonstrating differences within landmark across all three time intervals

Landmark	Critical value (χ^2^)	Significance^a^
Medial meniscus	8.773	.012
Lateral malleolus	14.25	.001
Lateral epicondyle	4.9	.086

^a^Significance level, .05; degrees of freedom, 2; *N* = 16

The *post-hoc* testing revealed a statistically significant reduction in distance from the landmark in medial meniscus before educational intervention versus immediately post-intervention (z = − 2.807, *p* = 0.005).The lateral malleolus also displayed a statistically significant reduction before educational intervention vs. immediately post-intervention and before educational intervention vs. 3 days post intervention (z = − 3.173, *p* = 0.002; z = − 2.642, *p* = 0.008, respectively). No significant difference in reduction of distance was found between medial meniscus before educational intervention vs. 3 days post intervention (z = − 0.140, *p* = 0.888) and for both of the lateral epicondyle instances (before educational intervention vs. immediately post-intervention z = − 2.218, *p* = 0.027; before educational intervention vs. 3 days post-intervention z = − 1.811, *p* = 0.070) (Table 3).

**Table 3:** Time comparison differences between pre- and post-intervention and pre-intervention and 3 day follow-up time intervals for each landmark (Post-hoc test)

Landmark	Times*	Critical value (Z)	*p*-value^ƚ^
Medial meniscus	1 vs. 2	−2.807	.005
	1 vs. 3	−.140	.888
Lateral malleolus	1 vs. 2	−3.173	.002
	1 vs. 3	−2.642	.008
Lateral epicondyle	1 vs. 2	−2.218	.027
	1 vs. 3	−1.811	.070

*Time 1 = First session, before educational intervention

*Time 2 = First session, immediate post-intervention

*Time 3 = Second session, 3 days post-intervention

^ƚ^significance value after Bonferroni adjustment is *p* ≤ .0083

## Discussion

Our results demonstrated significant differences at identifying the medial meniscus, lateral malleolus and the lateral epicondyle compared to US as the reference. Significant improvement was noted by the interns at identifying the medial meniscus at an immediate-post follow-up but not at a 3-day follow-up. This may be explained by the presence of the 3 outliers (35, 30 and 32 mm), where dramatic worsening performance by the interns was observed at the 3-day follow-up. In contrast, at the lateral malleolus, significant improvement was retained both at immediate-post and 3-day follow-ups. When taken as a whole, these results demonstrate significant discrepancy at identifying the medial meniscus, lateral malleolus and lateral epicondyle by the interns; however, with a brief 3-min educational US-guided intervention, the interns were able to accurately identify the medial meniscus and the lateral malleolus immediately post-follow up by identifying the landmarks on the contralateral side of the subject model. Lateral malleolus was the only landmark that the interns accurately identified on the 3-day follow-up. The quality of the 3-min educational US-guided intervention is unknown. More rigorous study design needs to be implemented into future studies to better understand the effect of the educational intervention.

There is no other study in the literature with which these results can be compared to, however, several studies were done in the spine assessing the accuracy of identifying spinal landmarks. In a systemic review and a meta-analysis, Cooperstein et al. addressed the location of the inferior angle of the scapula in relation to the upright spine. In chiropractic education, a commonly taught method is “7 up, 6 down,” referring to the position of the inferior angle of the scapulae in relation to the thoracic spinous process in upright and prone positions, respectively. In this article, there were 5 qualifying studies whose data were pooled, with the average spinous process level deemed to be T8, with a range of T4-T11 [7]. Anther common traditionally used landmark is using the intercrestal line to identify the L4 spinous process. Chakraverty et al. states L4 spinous process level is not accurately palpated and suggests using the intercrestal line to guide for L3 or L3–4 spinal level [4]. These studies suggest limitations of blinded approach to identifying topographical landmarks which opens opportunities for studies using US-guided intervention.

Our results demonstrate that a brief US-guided palpation intervention may improve interns’ ability to identify the medial meniscus and the lateral malleolus. Although a 3-day follow-up was an arbitrary interval, improvement was noted with identifying the lateral malleolus. In medicine, the implementation of US is emerging in teaching musculoskeletal anatomy [18–20] and physical diagnosis courses [21]. Numerous studies have demonstrated improvement in medical student confidence level [19] and scores [19, 22] with the implementation of the US in anatomy education. When anatomy learning was combined with the hands-on learning through US, being able to navigate through different planes of the anatomy, student engagement in learning anatomy was deemed promising [19]. As US-guided anatomy training is emerging in the medical curriculum, US-guided palpation intervention may have an impact for the palpatory education for the chiropractic students.

There are several limitations of the study. One is clearly its small sample size, which increases the probability of a type II error. However, this pilot study was intended to assess the feasibility to further the research in larger scale.

Another limitation of the study was the use of only 1 subject model. This subject model had a BMI of 19.5, which does not represent the general population. Even when the landmarks are covered by minute subcutaneous fat, discrepancy was yet noted across the medial meniscus and lateral malleolus, and it is safe to assume that the discrepancy will be larger with increase in the subject BMI. Future studies should include subjects with different BMI to represent the general population.

Another limitation is lack of a control group for comparison. Although having a control group is the preferred method, there is no known valid training for palpation in the literature comparable to ultrasound. Uncertainties of the ‘blinded’ educational intervention to the interns led the authors to omit a control group in the study. In the future, better understanding of the effect of the US-guided intervention needs to be evaluated through a larger sample longitudinal study design.

Finally, a convenient recruitment from a student body at a chiropractic institution does not represent the general chiropractic profession. However, the primary objective was to observe whether improvement is gained among the interns, and the US-guided palpation intervention may assist palpatory training in chiropractic programs.

For future investigation, a longitudinal study with a larger sample size should be analyzed to assess the effect of US-guided intervention on palpatory education.

## Conclusion

This pilot study demonstrated palpatory discrepancy between interns and US-guided palpation at identifying all three landmarks. Furthermore, US-guided palpation intervention improved interns’ ability of palpating 2 of the 3 landmarks (medial meniscus and lateral malleolus) following US-guided palpation intervention. US-guided palpation intervention may be a valuable addition to traditional palpatory education for chiropractic students if further larger sample studies demonstrate more benefit.

## Data Availability

The dataset collected and analyzed are available from the corresponding author upon request.

## Abbreviations

ATFL: Anterior talofibular ligament
RMSK: Registered in musculoskeletal sonography
US: Ultrasound
